# EGFR Mutation Analysis of Circulating Tumor DNA Using an Improved PNA-LNA PCR Clamp Method

**DOI:** 10.1155/2016/5297329

**Published:** 2016-07-13

**Authors:** Kana Watanabe, Tatsuro Fukuhara, Yoko Tsukita, Mami Morita, Aya Suzuki, Nobuyuki Tanaka, Hiroshi Terasaki, Toshihiro Nukiwa, Makoto Maemondo

**Affiliations:** ^1^Department of Respiratory Medicine, Miyagi Cancer Center, 47-1 Nodayama, Medeshima-Shiote, Natori 981-1293, Japan; ^2^Division of Cancer Biology and Therapeutics, Miyagi Cancer Center Research Institute, Natori, Japan; ^3^Molecular Genetic Research Department, LSI Medience Corporation, Tokyo, Japan; ^4^Japan Anti-Tuberculosis Association, Tokyo, Japan

## Abstract

*Introduction*. Rebiopsies have become more crucial in non-small cell lung cancer (NSCLC). Instead of invasive biopsies, development of collecting biological data of the tumor from blood samples is expected. We conducted a prospective study to assess the feasibility of detection of epidermal growth factor receptor (EGFR) mutation in plasma samples.* Method*. NSCLC patients harboring EGFR activating mutations, who were going to receive EGFR-tyrosine kinase inhibitors (TKIs) as first-line treatment, were enrolled in this study. Plasma EGFR activating mutations and the T790M resistance mutation were analyzed by an improved PNA-LNA PCR clamp method, characterized by a 10-fold or more sensitivity compared with the original methods.* Result*. Six patients with wild-type EGFR and 24 patients with EGFR mutations were enrolled in this study. Pretreatment plasma samples achieved sensitivity of 79%. The 6 patients with wild-type EGFR were all negative for plasma EGFR mutations. At the time of disease progression, plasma T790M mutation was detected in 8 of 16 cases. Absence of T790M before and during TKI treatment and disappearance of activating mutations during TKI treatment were considered as predictors of EGFR-TKIs efficacy.* Conclusion*. We were able to detect EGFR mutations in plasma samples by using an improved PNA-LNA PCR clamp method.

## 1. Introduction

EGFR mutations have become crucial predictors of the effect of epidermal growth factor receptor-tyrosine kinase inhibitors (EGFR-TKIs) in non-small cell lung cancer (NSCLC). Several reports demonstrated that gefitinib, erlotinib, and afatinib were significantly superior to chemotherapy in terms of efficacy [1–6]. First-line EGFR-TKIs have become standard treatments in NSCLC harboring EGFR mutations, substituting platinum doublet. Detection of driver mutations including EGFR mutations and anaplastic lymphoma kinase rearrangements has become a routine test at diagnosis as well as distinction of the histological type. One clinical issue is how to precisely detect driver mutations. Detection rate is influenced by various factors including size, fixation, preservation of samples, and extraction of DNA. Another issue related to EGFR mutation analysis is how to detect resistant EGFR mutations. First generation TKIs, gefitinib and erlotinib, cannot control disease progression for more than a year in numerous patients with EGFR mutations. More than 50% of mechanisms of resistance are due to the T790M mutation [7]. So far, detection of the T790M mutation availed just for negative selection. Recently, 3rd generation TKIs, effective on T790M mutated tumors, were developed and have become available in the United States. Consequently, it will be essential to confirm the existence of T790M mutations in tumor resistant to EGFR-TKIs. Though rebiopsies have been recommended to confirm EGFR mutation status, it is not always possible to obtain them because of the tumor size and the sites of primary or metastatic tumors. Therefore, less harmful and convenient procedures were desired.

A new approach to detect mutations in circulating tumor DNA (ctDNA) from plasma, so-called liquid biopsy, is being developed. Though liquid biopsies include ctDNA, circulating tumor cells, and exosomal RNA, the term liquid biopsy (liquid biopsy refers to collection of circulating tumor DNA in plasma in this report) was used referring to ctDNA in this study. Blood specimens are easy to obtain compared with cytological and histological samples, but previous reports have shown that detection rates of mutations in liquid biopsies were too low for clinical use [8]. There are several methods to detect plasma cell-free DNA, including high-sensitive PCR methods, digital PCR (dPCR), and next-generation sequencing (NGS). We have used a high-sensitive PCR method, PNA-LNA PCR clamp analysis, established by our group [9]. We have conducted several clinical studies for EGFR-mutated NSCLCs detected with this PNA-LNA clamp method [1, 10–12]. Since the original PNA-LNA PCR clamp method does not have high sensitivity for liquid biopsy, we developed an improved PNA-LNA PCR clamp method with higher detection rate which has become an appropriate method for liquid biopsy. We conducted a prospective study on the effect of first-generation EGFR-TKIs on EGFR mutations using the highly sensitive PNA-LNA PCR clamp analysis of plasma samples.

## 2. Methods

### 2.1. Patients

The prospective study was conducted to analyze EGFR mutations in plasma samples. We enrolled inoperable stage III or stage IV NSCLC patients harboring EGFR activating mutations who were supposed to receive EGFR-TKIs as first-line treatment. Other eligible criteria were the following: activating EGFR mutations (exon 19 deletion, L858R, and minor mutations) detected by PNA-LNA PCR clamp method, Eastern Cooperative Oncology Group (ECOG) performance status (PS) 0 to 2, and adequate organ function. Written informed consent for the use of blood samples was obtained from all patients. The study protocol was approved by the Ethical Review Committee of the Miyagi Cancer Center.

### 2.2. Plasma Sample Collection and EGFR Mutation Analysis

Whole blood samples (14 mL) were collected in ethylenediaminetetraacetic acid (EDTA) tubes before TKI treatment (P0), 2 months after the initiation of the treatment (P1), and after disease progression (P2). Samples were mixed thoroughly, and plasma isolated by centrifugation at 2000 ×g for 10 min was stored at −20°C. Collection of cytohistological samples was mandatory before treatment and was recommended after progression of disease (PD). DNA was extracted from plasma samples with QIAamp Circulating Nucleic Acid or from cytohistological samples with QIAamp DNA FFPE Tissue (QIAGEN, Hilden, Germany). PCR primers were designed to amplify G719X, exon 19 deletion, T790M, L858R, and L861Q. Here, we briefly explain mechanism of PNA-LNA clamp method (supplemental figure in Supplementary Material available online at http://dx.doi.org/10.1155/2016/5297329). This system utilizes PNA/LNA that binds complementary bases more firmly compared to DNA/DNA binding. PNA clamps were composed to be complementary to the respective wild-type alleles and LNA probes are constructed complementary to each mutation allele (Supplemental Table 1). Namely, PNA clamp hinders amplification of wild-type alleles and mutation sequences are effectively detected by LNA probe in real-time PCR cycling.

Samples were collected and stored at the Miyagi Cancer Center, and mutations were analyzed in the central laboratory, LSI Medience Corporation (Tokyo, Japan). PCR reaction conditions are shown in Supplemental Table 2. This improved PNA-LNA clamp method achieved less than 0.1% detection rate by using smaller PCR products and by increasing the number of cycles from 45 to 50 using a LightCycler 480 Instrument (Roche). Wild-type EGFR samples were also included in the mutation positive samples to determine the specificity of these analyses. Data of mutations obtained from cytohistological samples were blinded to investigators in the central laboratory to avoid biases.

### 2.3. Endpoints

We prospectively evaluated concordance rate of EGFR mutations between plasma samples and cytohistological samples using the improved PNA-LNA PCR clamp method and tried to determine the factors influencing detection rate of EGFR mutations in the plasma. The relation between the effect of EGFR-TKI and the status of EGFR activating mutations and T790M mutation in blood samples before and during EGFR-TKI treatment was also analyzed. This study focuses not only on T790M mutation but also on activating mutations; alternation of activating or resistance mutations during TKI treatment was assessed as a predictive factor of TKI treatment efficacy.

### 2.4. Statistical Analysis

The chi-square test was used to assess detection rate of* EGFR *mutations in the plasma among characteristics including intrathoracic or extrathoracic lesions. *P* values were two-tailed, and *P* < 0.01 was considered statistically significant. Progression-free survival (PFS) was defined as the time from the starting day of EGFR-TKI treatment. PFS and overall survival (OS) were calculated using the Kaplan-Meier method, and correlation between EGFR mutation status and effects of TKIs was performed by log-rank test. All the analyses were conducted using SPSS version 12 (IBM SPSS Statistics, IBM, Tokyo, Japan).

## 3. Results

### 3.1. Patients' Characteristics

A total of 24 lung adenocarcinoma patients met the enrollment criteria and participated in the study from December 2012 to March 2014. This study was performed at the Miyagi Cancer Center. Patients' characteristics are shown in Table 1. In summary, the median age was 67.0 years, 16 patients (66.7%) were female, 18 patients (75.0%) were never smokers, 22 patients (91.7%) had ECOG PS 0-1, and 22 patients (91.7%) presented with stage IV disease (8 patients with M1a and 14 patients with M1b metastases).

### 3.2. Correlation of EGFR Mutation Status between Tumor and Plasma Samples

The EGFR mutation status in tumors was initially analyzed by the conventional PNA-LNA PCR clamp method and the results are shown in Table 1. In 24 patients with EGFR mutations, 15 patients had exon 19 deletions, 8 patients had exon 21 L858R mutation, and 1 patient had exon 18 L861Q mutation. Furthermore, 6 patients with cytohistological wild-type EGFR were analyzed for plasma EGFR to evaluate the specificity of plasma EGFR mutation status. The results of the correlation of EGFR mutation status between tumor and baseline plasma samples are shown in Table 2. Of the 24 patients with EGFR-mutant tumors, 19 patients had detectable EGFR mutations in baseline plasma specimens with sensitivity of 79.2% and sensitivity for common mutations (exon 19 deletion and L858R mutation) of 82.4%. All mutation types in plasma-positive patients were consistent between plasma and tumor specimens. Of 6 patients with wild-type EGFR tumors, the plasma samples were all negative for EGFR mutations. Therefore, the specificity was 100%. As shown in Table 1, some factors including uncommon mutations and distal metastases might be correlated with the detection rate. Patients without extrathoracic metastases showed a significantly lower detection rate of plasma EGFR mutation than those with extrathoracic metastases (5/10 versus 14/14; *P* < 0.003). There was no significant difference between the detection rate of exon 19 deletion and L858R mutation.

### 3.3. Efficacy of EGFR-TKI Treatment In Patients with EGFR-Mutant Tumors

**Figure 1:**
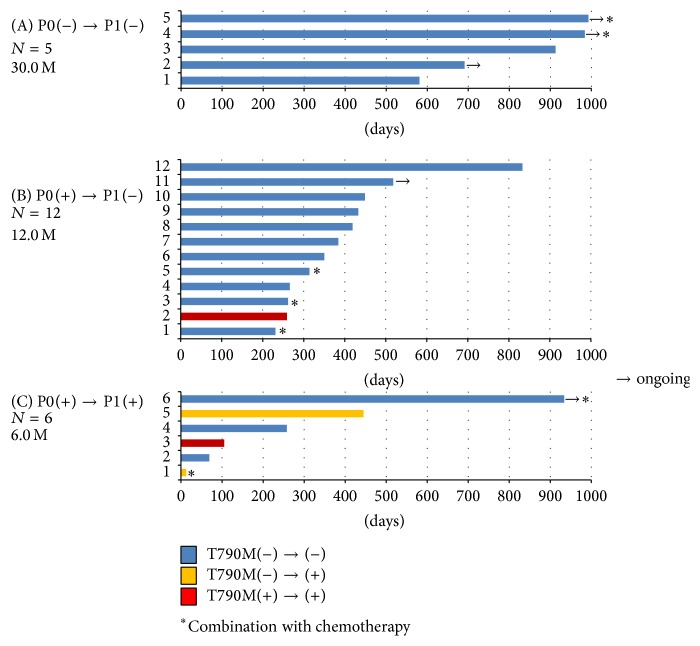
Relation between plasma EGFR mutations and duration of EGFR-TKIs treatment. (A): group of cases having no EGFR mutation in the plasma before TKI treatment (P0) and during TKI treatment (P1). (B): group of patients whose EGFR mutation status converted from positive at P0 to negative at P1. (C): group of patients with mutations both at P0 and at P1. Arrows indicate cases that are still under treatment. Asterisks show cases receiving combination of TKIs and chemotherapy.

**Figure 2:**
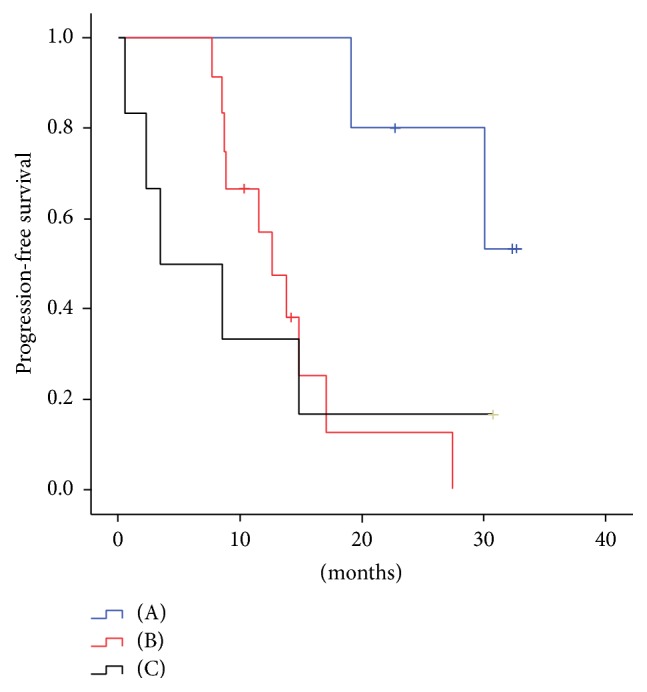
Kaplan-Meier curves for progression-free survival of the groups described in Figure 1.

All 24 patients with EGFR-mutant tumors received EGFR-TKI as first-line treatment: 23 received gefitinib and 1 received erlotinib. Twenty-three patients achieved partial response (PR) and 1 patient had stable disease. No patient achieved complete response. The objective response rate (ORR) and disease control rate (DCR) were 95.8% and 100%, respectively. There was no significant difference in ORR or DCR between patients with positive and negative baseline plasma EGFR mutations (94.7% versus 100% and 100% versus 100%, resp.). We evaluated the effect of treatment duration of TKIs, in addition to tumor response. Five cases negative for plasma EGFR mutations at baseline (P0) were still negative 2 months after initiation of the treatment (P1) (group A). Of 19 cases positive for plasma activating EGFR mutations at P0, 12 cases had no plasma mutations at P1 (group B) and 6 cases were still positive at P1 (group C) (Figure 1). In group C, the detected activating mutations were all identical to the baseline mutations. Patients negative for EGFR mutations had longer PFS, more than 30 months, with no median PFS (Figure 2). Three of five cases in group A were under treatment at cut-off point. Median treatment duration for groups B and C was 12 months and 6 months, respectively. There are no serious differences in characteristics among these three groups (Table 3). Because patients negative for plasma mutations tended to have localized tumors, long treatment duration was acceptable. The disappearance of EGFR mutations in the plasma samples of patients after TKI treatment might be a favorable predictive factor for response to EGFR-TKI treatment.

### 3.4. T790M Detection in the Plasma and Correlation with TKI Efficacy

At baseline, the drug resistance mutation T790M, a* de novo* mutation, was detected in 2 of 24 cases without the T790M mutation detected by conventional analyses in the tumor (Table 4). These 2 cases had short treatment duration compared with the T790M-negative cases at baseline. Detection of the* de novo *T790M mutation might be related to the high sensitivity of this analysis. At P1, T790M was newly detected in 2 cases. One case discontinued TKI treatment less than one month after initiation due to pneumotoxicity. The other case, having postoperative recurrence, underwent TKI treatment for more than a year. At disease progression (P2), T790M mutation was detected in 8 of 16 cases (50.0%) with sufficient frequency, and the activating mutation was observed in 11 of 16 cases (68.8%). Only 3 cases who could undergo rebiopsy at P2 had both the activating mutation and the T790M mutation detected in cytohistological as well as plasma samples. There was a complete match between plasma and cytohistological samples.

## 4. Discussion

This study showed that a high detection rate for EGFR mutations in the blood could be achieved by an improved PNA-LNA PCR clamp method. Results from plasma and cytohistological samples were approximately 80% concordant. Detection of mutations in the plasma of patients without extrathoracic metastases was harder than in patients with extrathoracic metastases. The disappearance of activating mutations during TKI treatment represents a candidate for new predictive factors for TKI treatment.

NGS or dPCR had attracted attention over the years as possible methods for liquid biopsy. However, these methodologies were expensive and the enormous amount of data obtained from NGS was difficult to manage. On the other hand, the improved PNA-LNA PCR clamp method could achieve high detection rate of EGFR mutations at low costs. Several recent meta-analyses showed 62–65% of sensitivity and 88–97% of specificity [13–16]. A clinically useful detection rate is supposed to be more than 80%, and our method approximately reached this value. Original PNA-LNA PCR clamp methods are commercially available in Japan, but their sensitivity is approximately 1%. We improved the sensitivity to 0.1% by changing primer sites and a thermal cycler. This method has advantages in the cost-benefit balance compared with dPCR and NGS. This PCR analysis costs about $200–300 for main activating and resistance mutations of a plasma specimen. Recently, Thress et al. reported comparisons among cobas EGFR mutation test, amplification refractory mutation system (ARMS)-PCR, droplet dPCR, and BEAMing dPCR in liquid biopsy [17]. Both droplet dPCR and BEAMing dPCR had the highest sensitivity in detecting T790M mutation, followed in order by cobas and ARMS-PCR. At the moment, digital platforms could be superior to nondigital platforms in terms of sensitivity, despite some false positive results obtained by digital platforms.

A second important advantage is that EGFR mutations can be detected in the blood. When liquid biopsies will be clinically available, they will be frequently used to avoid rebiopsies. If factors responsible for the inability to detect EGFR mutations will be elucidated, we will proceed to examine rebiopsies when we get negative results in liquid biopsy. In this study, the most critical factor was the site of the disease, restricted to the chest or not; on the basis of the TNM classification, this represents the distinction between M1b and the rest of the diseases. Activating mutations were observed in all patients with extrathoracic metastases, whereas they were observed in just 5 of 10 patients without extrathoracic metastases (50.0%). Some papers reported that it was hard to detect EGFR mutations from plasma samples of patients with intrathoracic disease [13, 18]. The other paper reported no difference among stages of the disease [19]. Since our data were consistent with these results, we have to keep in mind that it is difficult to get adequate results from liquid biopsies in patients with intrathoracic lesions including pulmonary metastases.

Another issue is the relation between the results of liquid biopsy and the effect of EGFR-TKI. The factor influencing PFS was the absence of any EGFR mutation in the plasma before treatment. As we mentioned above, this factor was only associated with patient with intrathoracic diseases. Intrathoracic distribution of disease without extrathoracic metastases might be a predictive factor for long PFS. Another critical factor is the disappearance of activating mutations in the plasma during EGFR-TKI treatment, which could be closely related to the efficacy of EGFR-TKIs. However, persistent existence of the EGFR T790M mutation, the activating mutation, or both in the plasma, despite TKI treatment, was an unfavorable factor related to short PFS. Tseng et al. showed that cases with persistently detectable mutations in the plasma had shorter PFS and OS than cases with undetectable plasma EGFR mutations at baseline and cases with mutations in the plasma disappearing after EGFR-TKI treatment [18]. This previous report demonstrated that cases with undetectable plasma mutations had longer survival than cases where the mutations disappeared, despite no difference in terms of PFS. Here we emphasize that disappearance of activating mutations in the plasma could be a predictive factor for EGFR-TKI efficacy in addition to the absence of plasma mutations at baseline.

This study has several limitations, including the small sample size and the enrollment of patients from a single institute. Now we are expanding our study collecting samples from several institutes using our highly sensitive method and planning to compare this method to digital PCR or NGS.

In conclusion, we were able to effectively detect EGFR activating or drug-resistant mutations in blood samples using an improved PNA-LNA PCR clamp method, which is inexpensive and accessible compared with dPCR or next-generation DNA sequencing.

## Supplementary Material

Supplemental Figure. Mechanism of PNA-LNA Clamp PCR.
Supplemental Table 1. Primers and probes listed by assay.
Supplemental Table 2. PCR cycles.

## Figures and Tables

**Table 1:** Patients' characteristics.

	EGFR mutant (biopsy sample)	EGFR mutant (biopsy sample)		Wild-type EGFR (biopsy sample)
		Plasma *EGFR* mut+	Plasma *EGFR* mut−	
Total, *n*	24	19	5	6
Age				
Median (range)	67 (46–87)	67 (46–87)	68 (55–84)	70 (64–79)
Gender				
Female	16 (66.7)	11 (57.9)	5 (100)	1 (16.7)
Male	8 (33.3)	8 (42.1)	0 (0.0)	5 (83.3)
Smoking status				
Never	18 (75%)	14 (73.7)	4 (80.0)	2 (33.3)
Former	3 (12.5)	2 (10.5)	1 (20.0)	1 (16.7)
Current	3 (12.5)	3 (15.8)	0 (0.0)	3 (50.0)
ECOG PS				
0	8 (33.3)	5 (26.3)	3 (60.0)	1 (16.7)
1	14 (58.3)	12 (63.2)	2 (40.0)	5 (83.3)
2	2 (8.3)	2 (10.5)	0 (0.0)	0 (0.0)
Stage				
IIA	0 (0.0)	0 (0.0)	0 (0.0)	1 (16.7)
IIIA	2 (8.3)	1 (5.3)	1 (20.0)	1 (16.7)
IV	22 (91.7)	18 (94.7)	4 (80.0)	4 (66.7)
IV-M1a	8 (36.4)	4 (22.2)	4 (100)	1 (16.7)
IV-M1b	14 (63.6)	14 (77.8)	0 (0.0)	3 (50.0)
Tumor EGFR mutation status				
Wild type	0	0	0	6 (100)
del19	15 (62.5)	12 (63.2)	3 (60.0)	0
L858R	8 (33.3)	7 (36.8)	1 (20.0)	0
L861Q	1 (4.2)	0 (0.0)	1 (20.0)	0

**Table 2:** Correlation of EGFR mutation status between tissue and plasma samples before EGFR-TKI treatment.

	Pretreatment plasma: P0		Total
	Positive	Negative	
Pretreatment tissue			
Positive	19	5	24
Negative	0	6	6

Total	19	11	30

Sensitivity, 79.2%; specificity, 100%.

**Table 3:** Detection of EGFR mutations from tissue and plasma samples.

*N* (%)	Tissue	Plasma			Tissue
	Before treatment	Before treatment P0 (%)	Under treatment P1 (%)	After PD P2 (%)	After PD
Number of samples	24	24	23	16	3
Activating mutation	24	19 (79.2)	6 (26.1)	11 (68.8)	3 (100)
Drug-resistant mutation (T790M)	NA	2 (8.3)	4 (17.4)	8 (50.0)	3 (100)

**Table 4:** Characteristics of patients with alteration of the EGFR mutation status after EGFR-TKI treatment.

	Persistent plasma EGFR mutation negative (A)	Conversion of plasma EGFR mutation from positive to negative (B)	Persistent plasma EGFR mutation positive (C)
Total, *n*	5	12	6
Age			
Median (range)	68 (55–84)	65.5 (46–87)	66.5 (58–79)
Gender			
Female	5 (100)	7 (58.3)	3 (50.0)
Male	0 (0.0)	5 (41.7)	3 (50.0)
Smoking status			
Never	4 (80.0)	9 (75.0)	4 (66.6)
Former	1 (20.0)	1 (8.3)	1 (16.7)
Current	0 (0.0)	2 (16.7)	1 (16.7)
ECOG PS			
0	3 (60.0)	4 (33.3)	0 (0.0)
1	2 (40.0)	7 (58.3)	5 (83.3)
2	0 (0.0)	1 (8.3)	1 (16.7)
Stage			
IIIA	1 (20.0)	1 (8.3)	0 (0.0)
IV	4 (80.0)	11 (91.7)	6 (100)
IV-M1a	4 (100)	4 (36.4)	0 (0.0)
IV-M1b	0 (0.0)	7 (63.6)	6 (100)
Tumor EGFR mutation status			
del19	3 (60.0)	8 (66.7)	3 (50.0)
L858R	1 (20.0)	4 (33.3)	3 (50.0)
L861Q	1 (20.0)	0 (0.0)	0 (0.0)

## Abbreviations

ctDNA: Circulating tumor DNA
ECOG: Eastern Cooperative Oncology Group
EDTA: Ethylenediaminetetraacetic acid
EGFR: Epidermal growth factor receptor
DCR: Disease control rate
dPCR: Digital PCR
NGS: Next-generation sequencing
NSCLC: Non-small cell lung cancer
OS: Overall survival
ORR: Objective response rate
PFS: Progression-free survival
PNA-LNA PCR clamp method: Peptide nucleic acid-locked nucleic acid polymerase chain reaction clamp method
PR: Partial response
PS: Performance status
TKIs: Tyrosine kinase inhibitors.
