# Supplementary material for: EGFR Mutation Analysis of Circulating Tumor DNA Using an Improved PNA-LNA PCR Clamp Method
Source: Can Respir J. 2016 Jul 13;2016:5297329. doi: 10.1155/2016/5297329 (PMC4961805; doi:10.1155/2016/5297329)
Supplement: Supplementary file 1 — Supplemental Figure. Mechanism of PNA-LNA Clamp PCR. Supplemental Table 1. Primers and probes listed by assay. Supplemental Table 2. PCR cycles. [file 5297329.f1.pdf]

Supplemental Figure. Mechanism of PNA-LNA Clamp PCR

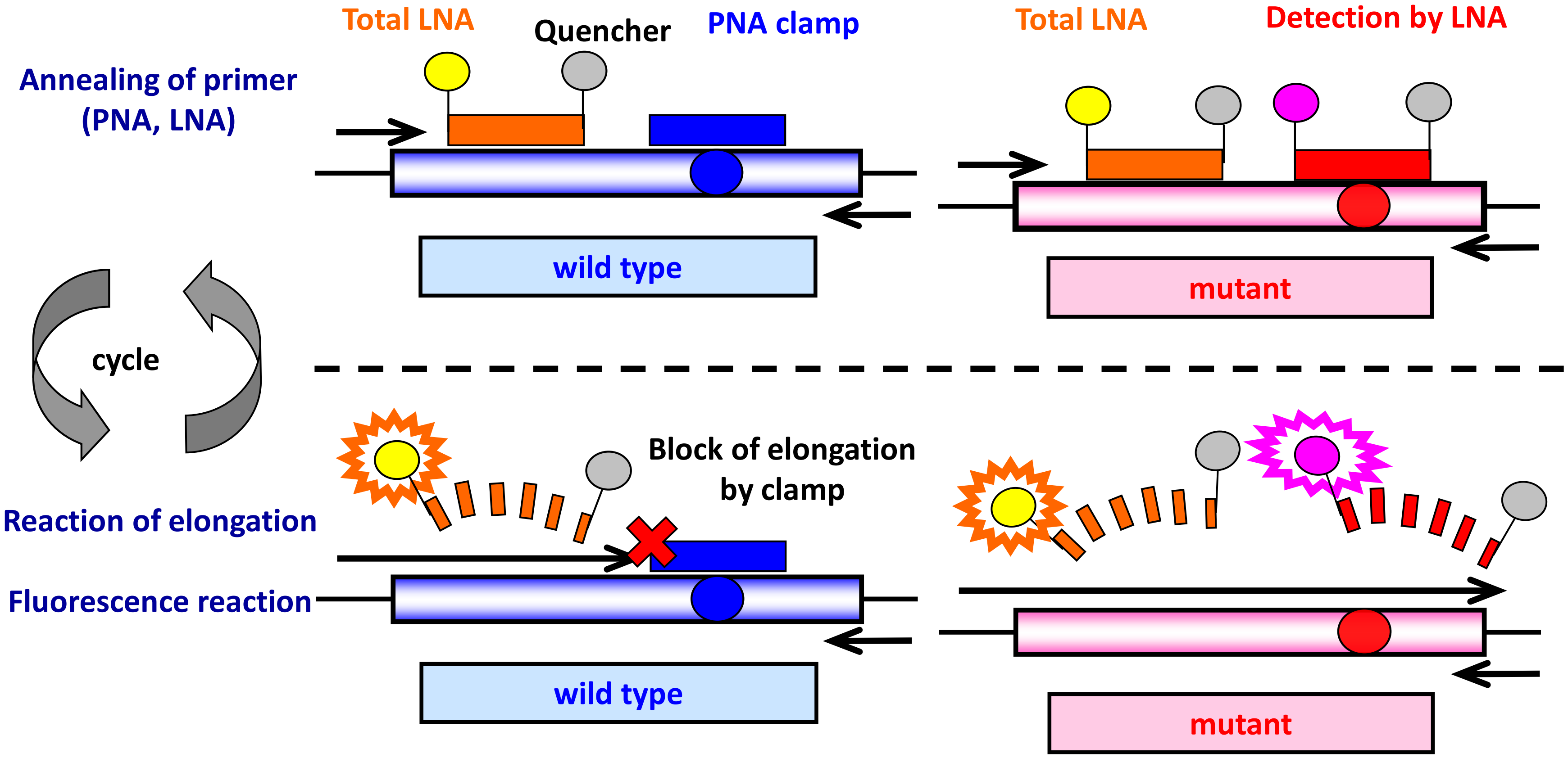

- Blocking amplification of wild type allele by PNA
- Specific detection of mutant alleleby LNA

Supplemental Table 1. Primers and probes listed by assay.

	Oligo name	Oligo Sequence 5'→3'	Final conc. (nM)
Reaction1 (ex18 G719S, G719A, G719C)			
PCR primer	ex18-F	5'-CTTACACCCAGTGGAGAAGC-3'	200
	ex18-R	5'-GGACCTTACCTTATACACCG-3'	200
Mutation Probe	ex18-LNA-G719S	5'-Cy3/ACCGGAGC+TCAGCAC/BHQ-3'	100
	ex18-LNA-G719A	5'-FAM/CGGAG+G+CCAGC+A/BHQ-3'	100
	ex18-LNA-G719C	5'-FAM/ACCGGAGC+ACAGCAC/BHQ-3'	100
Total Probe	ex18-Total	5'-Cy5/ CAGTTTC+CTT+CAAGA+TCCTC/BHQ-3'	100
PNA	ex18- PNA	N- GAGCCCAGCACTTT -C	500
Reaction2 (ex19 E746-A750(2235-2249)del , E746-A750(2236-2250)del)			
PCR primer	ex19-F	5'-TGTCATAGGGACTCTGGATCC-3'	200
	ex19-R	5'-AGCAGAAACTCACATCGAG-3'	200
Mutation Probe	ex19-E746-A750(2235-2249)del-LNA	5'-FAM/CTATCAA+AA+CATCTCCGAAAGC/BHQ-3'	100
	ex19-E746-A750(2236-2250)del-LNA	5'-Cy3/CTATCAA+GA+CATCTCCGAAAGC/BHQ-3'	100
Total Probe	ex19-Total	5'-5Cy5/TTAA+CT+T+T+CT+CA+CCT/3BHQ-3'	100
PNA	ex19 PNA	N- AGATGTTGCTTCTCTTAA- C	2500
Reaction3 (ex20 T790M)			
PCR primer	ex20-F	5'-GCCTCACCTCCACCGTGC-3'	200
	ex20-R	5'-TGTTCCCGGACATAGTCCAG-3'	200
Mutation Probe	ex20-LNA-T790M	5'-FAM/CTCATCA+TGCAGCTCATG/BHQ-3'	100
Total Probe	ex20-Total	5'-Cy5/CTT+CGGC+TGC+CTC/BHQ-3'	100
PNA	Ex20 PNA	N- CTCATCACGCAGCTCA- C	500
Reaction4 (ex21 L858R)			
PCR primer	ex21-F	5'-GAACGTACTGGTGAAAACACC-3'	200
	ex21-R	5'-GCATGGTATTCTTTCTCTTCC-3'	200
Mutation Probe	ex21-LNA-L858R	5'- FAM/TTTGGCC+CGCCCAA/BHQ-3'	100
Total Probe	ex21-Total	5'-Cy5/CAGCATGT+CAAGA+TCACAGA/BHQ-3'	100
PNA	Ex21 PNA (R4)	N- TTTGGCCAGCCCAA- C	500
Reaction5 (ex21 L861Q)			
PCR primer	ex21-F	5'-GAACGTACTGGTGAAAACACC-3'	200
	ex21-R	5'-GCATGGTATTCTTTCTCTTCC-3'	200
Mutation Probe	ex21-LNA-L861Q	5'-TET/CACCCAGC+TGTTTGGC/BHQ-3'	100
Total Probe	ex21-Total	5'-Cy5/CAGCATGT+CAAGA+TCACAGA/BHQ-3'	100
PNA	Ex21 PNA (R5)	N- ACCCAGCAGTTTGGC- C	500

Supplemental Table 2. PCR cycles

	Stage1		Stage2				
**Reaction name**	**Temp**	**sec**	**Temp**	**sec**	**Temp**	**sec**	**Cycles**
1, 2	95	10	95	3	62	30	50
3	95	10	95	3	58	30	50
4, 5	95	10	95	3	56	30	50
